# Perioperative tislelizumab with four cycles of neoadjuvant chemotherapy for resectable locally advanced esophageal squamous cell carcinoma: a phase 2 study

**DOI:** 10.3389/fimmu.2024.1482005

**Published:** 2024-12-02

**Authors:** Na Zhou, Yuwei Hua, Yuping Ge, Qiang Wang, Chenyu Wang, Jia He, Luo Zhao, Shuangni Yu, Junfang Yan, Lin Zhao, Li Li, Chunmei Bai

**Affiliations:** ^1^ Department of Medical Oncology, Peking Union Medical College Hospital, Beijing, China; ^2^ Department of Gastroenterology, Peking Union Medical College Hospital, Beijing, China; ^3^ Department of Thoracic Surgery, Peking Union Medical College Hospital, Beijing, China; ^4^ Department of Pathology, Peking Union Medical College Hospital, Beijing, China; ^5^ Department of Radiation Oncology, Peking Union Medical College Hospital, Beijing, China

**Keywords:** esophageal squamous cell carcinoma, perioperative treatment, tislelizumab, immunotherapy, neoadjuvant chemotherapy

## Abstract

**Background:**

The application of neoadjuvant immunotherapy in the treatment of esophageal cancer needs further exploration. This study aimed to investigate the safety and effectiveness of tislelizumab, an anti-PD-1 antibody, combined with chemotherapy as neoadjuvant treatment for locally advanced esophageal squamous cell carcinoma (LA-ESCC).

**Methods:**

In this phase II study, patients with clinical stages of II-IVA (T3-T4 and/or node positive) potentially resectable LA-ESCC were enrolled. Patients received neoadjuvant tislelizumab and chemotherapy every 3 weeks for 4 cycles before surgery and adjuvant tislelizumab for 9 months. The primary endpoint was pathological complete response (pCR) rate. Secondary endpoints included R0 resection, disease free survival (DFS), adverse events (AE), and biomarkers for predicting efficacy.

**Results:**

The study included 30 patients. 25 patients completed neoadjuvant chemoimmunotherapy and underwent surgery, 96% with R0 resection. The pCR and MPR rate was 44% and 52%. The 6-month and 1-year DFS rate was 100% and 75.3%. 43.3% patients experienced severe (grade 3-4) treatment-related adverse events (TRAEs) and 5 patients developed severe immune-related adverse events (irAEs). Further exploration found that a group of peripheral lymphocyte subsets increased significantly after 2 cycles of neoadjuvant therapy in patients who achieved pCR, suggesting the importance of dynamic monitoring of circulating lymphocyte.

**Conclusions:**

The combination of perioperative tislelizumab and neoadjuvant chemotherapy has achieved an encouraging pCR rate and demonstrated a manageable safety profile in patients with potentially resectable ESCC.

**Clinical Trial Registration:**

https://www.chictr.org.cn/, identifier ChiCTR2100043772.

## 1 Introduction

Esophageal cancer is one of the most common digestive tract tumors. It ranks 7th in incidence and 6th in mortality among all malignancies globally. The highest incidence and mortality of esophageal cancer are found in East Asia, where esophageal squamous cell carcinoma (ESCC) accounts for approximately 90% of all cases (1, 2).

Neoadjuvant chemotherapy (nCT) or neoadjuvant chemoradiotherapy (nCRT) both have been considered as the standard clinical managements for patients with locally advanced (LA), potentially resectable esophageal cancer in China before the era of immunotherapy. Based on JCOG 1109 trial, ESCC patients treated with preoperative chemotherapy using cisplatin plus 5-fluorouracil (CF) had no significantly difference in progression-free survival (PFS) and overall survival (OS) than those treated with nCRT using the same combination, while the addition of docetaxel to CF, the DCF regimen, significantly improved OS over the traditional doublet in patients with locally advanced ESCCs. However, pathological complete response (pCR) rate and R0 resection rate were relatively low in nCT and already hit the bottleneck (3). Moreover, the landmark CROSS study found that nCRT using taxane- and platinum-based regimen followed by surgery can confer higher pCR rate and survival benefit in LA-ESCCs (4, 5). Yet nearly half (49%) of patients in the nCRT group experience recurrence after surgery, and the 5-year survival rate is approximately 47% (4). Therefore, regimens that lead to rapid and deep tumor remission as well as long-term survival of patients are still needed.

Immunotherapy using immune checkpoint inhibitors (ICIs) has revolutionized the treatment landscape for various malignancies. Esophageal cancer is highlighted as a type of tumor with considerable mutation burden, comparable to non-small cell lung cancer (NSCLC) and melanoma (6). Multiple studies have repeatedly confirmed the effectiveness of the combinations of ICIs with platinum-based doublets as the upfront management for locally unresectable and metastatic ESCCs (7, 8). RATIONALE-306, a global, randomized, placebo-controlled, phase 3 study, revealed that the addition of Tislelizumab, a humanized IgG4 anti-PD-1 monoclonal antibody raised objective response rate by 21% over chemotherapy alone, regardless of PD-L1 expression levels (9). Besides, adjuvant immunotherapy of nivolumab has doubled the median disease-free survival (DFS) for resectable ESCC patients who had residual pathological disease after nCRT (10). Therefore, it’s reasonable to integrate anti-PD-1 antibodies as a “sandwich” therapy with traditional perioperative treatment of ESCCs since they may provide extra responses and survival benefits.

Here, we conducted an open-label, single-arm, phase 2 trial, aiming to investigate the efficacy and safety of tislelizumab plus taxane and platinum combined chemotherapy as a neoadjuvant treatment for locally advanced ESCC. Feasibility of tislelizumab as an adjuvant therapy following neoadjuvant chemoimmunotherapy and surgery was also explored.

## 2 Materials and methods

### 2.1 Protocol

This is a prospective, phase II, single arm study undertaken at the Peking Union Medical College Hospital, Beijing. (Registry number: ChiCTR2100043772) It received approval from the Ethics Committee at Cancer Hospital, Chinese Academy of Medical Sciences and Peking Union Medical College, China. The study was designed and conducted in compliance with all relevant regulations regarding the human research participants and followed the principles of the Helsinki Declaration. All patients provided written informed consent.

### 2.2 Participants

Participants eligible for enrollment were individuals aged 18 years or older with a histologically confirmed diagnosis of thoracic esophageal squamous cell carcinoma who had not received prior anticancer therapy. Only patients with tumors of clinical stage II-IVA according to the 8th edition of the American Joint Committee on Cancer (AJCC) TNM staging system were enrolled. Their tumors were deemed potentially resectable by an experienced multidisciplinary team (MDT). All participants had a World Health Organization (WHO) performance status score of 0-1 and adequate organ function.

### 2.3 Procedure

In the initial phase, participants received four cycles of neoadjuvant therapy, administered every three weeks. Each cycle includes docetaxel at 75mg/m^2^ intravenously on Day 1, cisplatin at 75mg/m^2^ intravenously on Day 1, and tislelizumab intravenously at 200mg/m^2^ on Day 1. For patients with renal insufficiency (creatinine clearance, CCr < 60ml/min), cisplatin can be changed to carboplatin with AUC=5. Imaging examinations were conducted after every two cycles, and changes in tumor size were evaluated following response evaluation criteria in solid tumors (RECIST) version 1.1 (11). Patients exhibiting distant metastasis during this stage were promptly excluded from the study, while those showing no signs of progress proceeded to the subsequent course of treatment. Surgery was performed 4 to 8 weeks after neoadjuvant therapy by experienced thoracic surgeons in our institute. McKeown minimally invasive esophagectomy (MIE) or Ivor Lewis esophagectomy with at least two-field lymphadenectomy were performed. After surgery, the regimen of adjuvant therapy was determined based on postoperative pathological staging, patient’s personal wishes, and clinicians’ evaluation. Follow-up was conducted every 12 weeks for the first 2 years. Subsequently, the visit interval was extended to every 6 months until 5 years after treatment.

Surgical specimens were evaluated by professional pathologists and pathological response was evaluated according to the tumor regression grade (TRG) score based on the College of American Pathologists (CAP) and American Joint Committee on Cancer(AJCC) (12), with TRG classified as 0 (complete remission, no viable cancer cells), 1 (near complete remission, single cells or rare small groups of cancer cells), 2 (partial remission, more than single cells or rare small groups of cancer cells with evident tumor regression), or 3 (poor or no response, extensive residual tumor or no regression). Pathological complete response (pCR) was defined as the absence of any residual tumor cells or only carcinoma *in situ* (ypT0/Tis N0) in the surgical specimen, while MPR (major pathological response) was defined as the presence of residual cancer cells at a level of ≤10%.

Adverse events were closely monitored according to the Common Terminology Criteria for Adverse Events (CTCAE) (version 5.0) guidelines. Surgical complications were assessed by Clavien-Dindo classification (13). In addition, baseline peripheral blood samples were collected, as well as samples after 2 and 4 cycles of neoadjuvant therapy, and the T cell subpopulations were assessed using flow cytometry.

### 2.4 Outcome

The primary endpoint was pCR rate. The secondary endpoints included R0 resection (defined as no cancer cells seen microscopically at the resection margin following surgery), disease free survival (DFS) and safety. DFS was defined as the time from radical resection to the recurrence or metastasis of the tumor, or death from any cause.

### 2.5 Statistics

The estimated sample size was 30 patients, assuming a null pCR proportion of 10% and an alternative pCR proportion of 20% (α = 0.05, 1-β = 0.80). 10% drop-out during follow-up was considered. The Shapiro–Wilk test was used to evaluate whether continuous variables were normally distributed. Continuous variables with normal distribution were expressed as mean ± standard deviation, and those with non-normal distribution were expressed as median and quartiles. Categorical variables were expressed as counts and percentages. The Kaplan–Meier method was used to estimate survival. When comparing between groups, the t test was used for normally distributed data and the Kruskal–Wallis test was used for non-normally distributed data. Fisher’s exact test was used for testing of categorical factors. Univariate and multivariate logistic regression were used to explore factors that predict prognosis. All tests were two sided, and a p<0.05 was considered significant. IBM SPSS (version 29) and R (version 4.3.0) within R Studio (version 2023.03.1.446) was used for statistical analysis. Figures were drawn using R Studio and GraphPad Prism (version 10.1.0).

## 3 Results

### 3.1 The clinicopathological characteristics of patients with LA-ESCCs at baseline

From Nov 11^th^, 2020, to Feb 10^th^, 2023, 30 Chinese patients diagnosed with locally advanced, thoracic ESCCs were enrolled in this study. The baseline demographic and clinicopathological characteristics of the patients were shown in **Table 1**. The median age of all patients was 60 years. Most patients were male (25/30, 83.3%). Half of the tumors located at the lower thoracic esophagus while the median tumor length was 5.5cm (range, 2.8-13.0cm).14 patients (46.7%) had clinical stage III ESCCs, whereas 16 (53.3%) had stage IVA diseases. It is noteworthy that more than 90% of patients in our study had T3 or T4 diseases and all had regional lymph node metastasis (cN+). The baseline staging was considered accurate since comprehensive imaging tools including contrast enhanced CT (100%), PET/CT (73.3%), endoscopic ultrasound (EUS) (80%) or their combinations were applied, and a multidisciplinary team (MDT) was convened to go through every single cases.

**Table 1:** Baseline characteristics.

n=30		
Age (yr), median (range)		60 (50-74)
Sex, n (%)		
	Female	5 (16.7)
	Male	25 (83.3)
Location, n (%)		
	Upper	5 (16.7)
	Middle	10 (33.3)
	Lower	15 (50.0)
Length (cm), median (range)		5.5 (2.8-13.0)
T stage, n (%)		
	T2	2 (6.7)
	T3	17 (56.7)
	T4	11 (36.7)
N stage, n (%)		
	N1	11 (36.7)
	N2	12 (40.0)
	N3	7 (23.3)
Stage, n (%)		
	III	14 (46.7)
	IVA	16 (53.3)
Grade, n (%)		
	G1	2 (6.7)
	G2	18 (60.0)
	G3	6 (20.0)
	Gx	4 (13.3)
Smoke status, n (%)		
	No	9 (30%)
	Yes	21 (70%)
Drink status, n (%)		
	No	4 (13.3%)
	Yes	26 (86.7)

### 3.2 Treatment exposure

**Figure 1:**
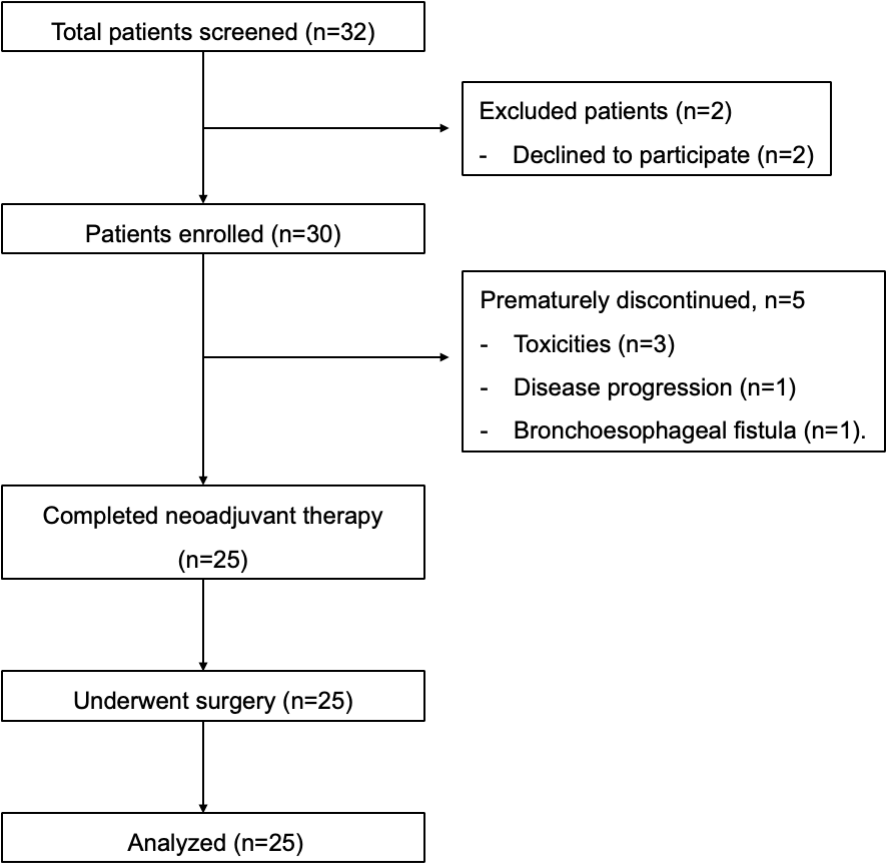
Consort diagram.

Consort diagram was shown in **Figure 1**. Out of the 30 LA-ESCC patients enrolled, 25 completed neoadjuvant chemoimmunotherapy (22 finished 4 cycles, and 3 finished 3 cycles) and underwent minimally invasive Ivor-Lewis or Mckeown esophagectomy afterwards. The mean dosage intensities were 94.97% (78.90%, 100%) for the first two cycles and 93.88% (81.60%, 100%) for the following cycles in neoadjuvant section. 6 patients swapped cisplatin for carboplatin during neoadjuvant section since their creatinine clearance levels dropped down to 50ml/min or below. The dosage of tislelizumab was never reduced or canceled in any of these patients. The reasons for early dropouts (n=5) before surgery were intolerable toxicities (n=3), disease progression at first evaluation (n=1) and bronchoesophageal fistula following the first cycle of treatment (n=1). Most patients (22/25) underwent surgery as planned. 3 patients were unable to undergo surgery on time, with 2 due to adverse events and 1 for personal reasons.

After surgery, 64% (16/25) patients continued adjuvant immunotherapy with the median cycles of 8. Among them, 7(43.8%) patients achieved pCR. 32% (8/25) patients received adjuvant radiotherapy for regional lymph nodes involvement after MDT discussion, 5 of them received tislelizumab after radiotherapy. The main reasons for refusing adjuvant tislelizumab were lack of efficacy in neoadjuvant setting (n=4) and patients’ own choices (n=5).

### 3.3 The efficacy of neoadjuvant chemoimmunotherapy and survival

Among 25 patients who underwent esophagectomy, 24 patients (96%) got R0 resection and 11 (44%) achieved a pCR (ypT0/Tis ypN0) both in primary tumor and lymph nodes. 1 patient achieved complete responses in primary lesion, however, had residual cancer in resected lymph nodes. The major pathological response (MPR) rate was 52%. As for TRG grading, 11 cases (44%) were categorized as grade 0, 2 (8%) as grade 1, 7 (24%) as grade 2 and 5 (24%) as grade 3 in primary tumor.

**Figure 2:**
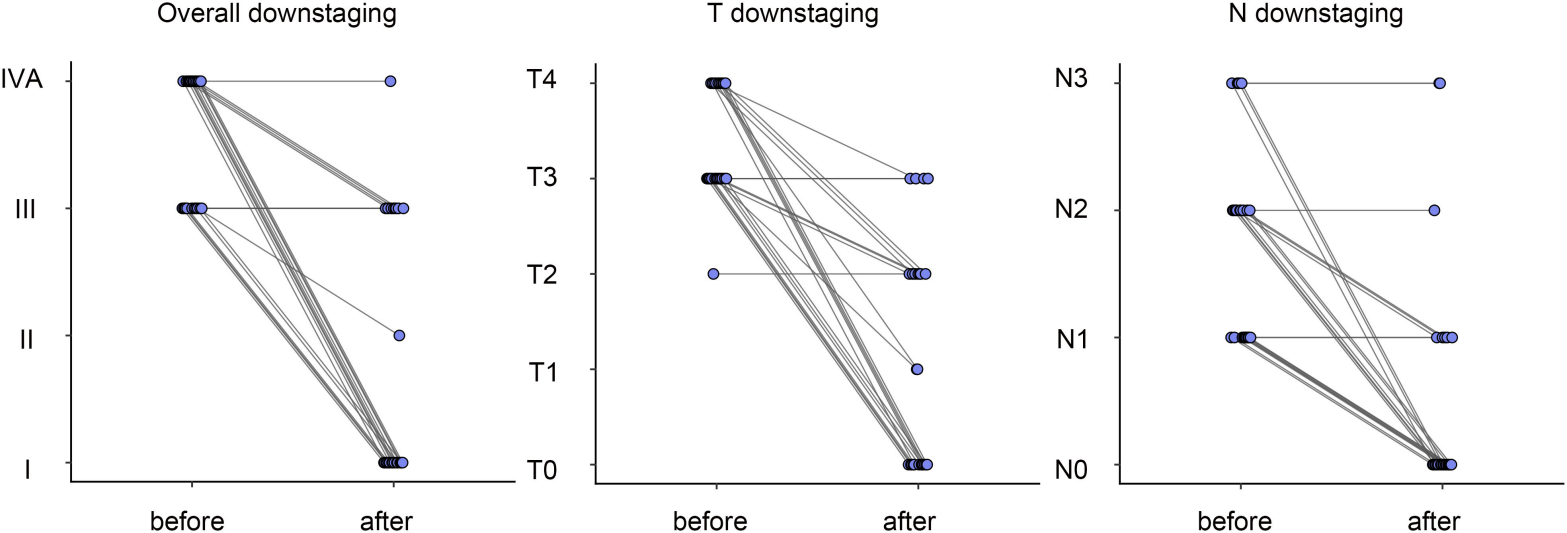
Changes of overall tumor staging, T staging and N staging before and after neoadjuvnat treatment according to the TNM classification.

As shown in **Figure 2**, 76% (19/25) of the patients achieved overall TNM downstaging. 21(84%) patients experienced shrinkage of the primary tumor, resulting in T stage downstaging, while 20 (80%) showed downstaging of lymph node metastasis. Notably, 68% (17/25) of patients showed node-negative after neoadjuvant treatments. Most patients (18/22) exhibited an early response to treatment after two cycles; however, none achieved complete response (CR). Among these patients, 50% attained pCR after competing four cycles of treatment.

**Figure 3:**
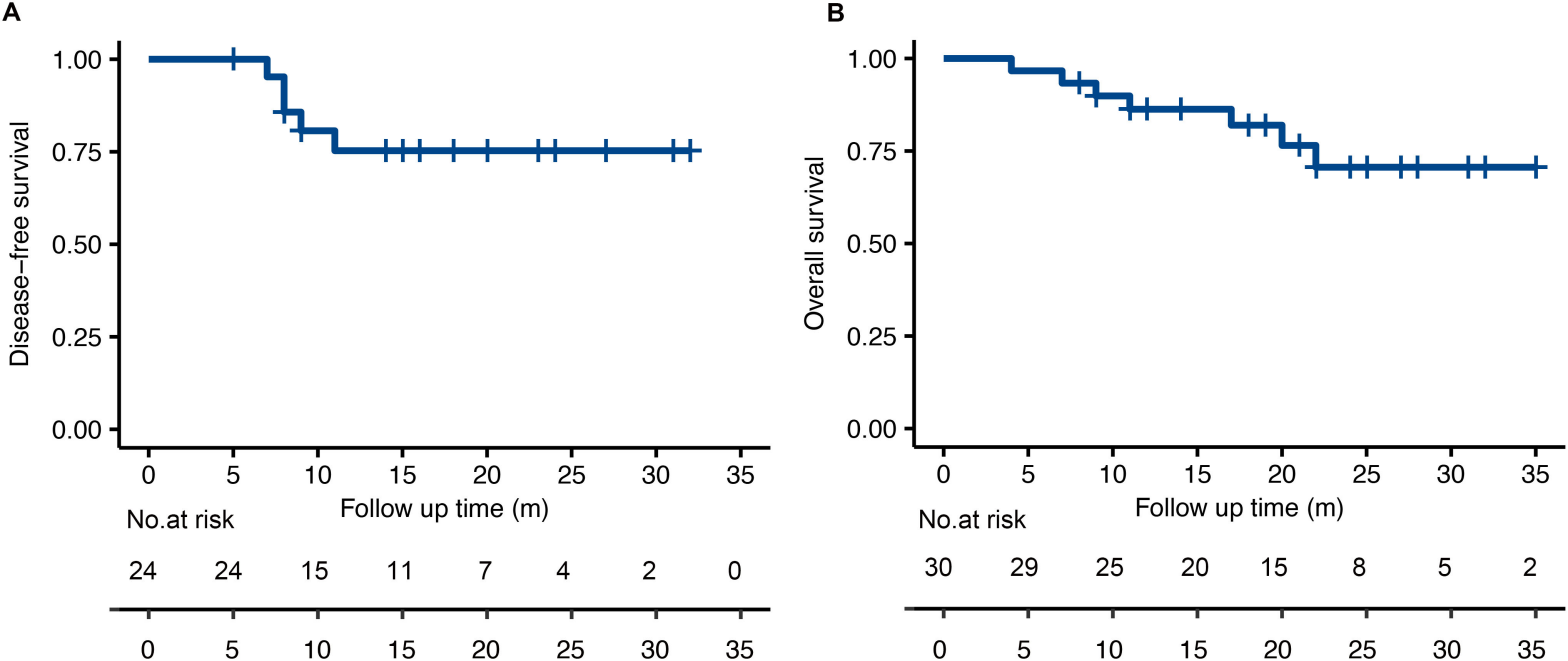
The Kaplan-Meier analysis for disease-free survival **(A)** and overall survival **(B)** in the treated population.

At the time of analysis (November 2023), the median follow-up time was 19.6 months (4-35 months). No patient was lost to follow-up. The survival curve was shown in **Figure 3**. The median DFS and the median OS was not reached by the end of the follow-up period. The 6-month and 1-year DFS was 100% and 75.3%. The 6-month and 1-year OS was 96.7% and 86.3%. 5 patients showed disease progression by the time of the last follow-up, 4 with distant metastases and 1 with locoregional progression. All distant metastases occurred in patients who didn’t achieve tumor downstaging and who had N2 or N3 diseases after neoadjuvant chemoimmunotherapy and surgery. The most common sites of distant metastases were lung (n=2), non-regional lymph nodes (n=2) and bones (n=1).

### 3.4 Safety

Most patients (80%, 24/30) experienced at least one treatment-related adverse events (TRAEs) (**Supplementary Table 1**), and most of the TRAEs were grade 1-2. The most common grade 1–2 TRAEs were creatine increase (26.7%), nausea (16.7%), rash (10%) and diarrhea (10%). 13 (43.3%) patients experienced severe TRAEs (grade 3-4) and most common severe TRAEs were neutropenia (30%). No treatment-related death was observed.

In terms of autoimmune toxicity, 9/30 (30%) patients experienced explicit immune-related adverse events (irAEs) during neoadjuvant treatment. (Shown in **Table 2**) The most common irAEs were skin toxicity (n=4, 13.3%), interstitial nephritis (n=4, 13.3%), thyrotoxicosis (n=2, 6.7%), hypophysitis (n=1, 3.3%), elevated transaminitis and bilirubin (n=1, 3.3%), elevation of amylase and lipase (n=1, 3.3%). No patients reported interstitial pneumonia. 4 patients (13.3%) had irAEs involving more than one system. Severe irAEs (grade 3-4) were observed in 4 patients who were treated with systematic corticosteroid with or without immunosuppressors. One patient who suffered from both hepatic and renal adverse recovered from bioartificial liver treatment.

**Table 2:** Immune-related adverse events.

Adverse events, n (%)	Grade 1/2	Grade 3/4	Total
Neoadjuvant phase			
Skin toxicity	2 (6.7%)	2 (6.7%)	4 (13.3%)
Interstitial nephritis	2 (6.7%)	2 (6.7%)	4 (13.3%)
Thyrotoxicosis	2 (6.7%)	0	2 (6.7%)
Hypophysitis	1 (3.3%)	0	1 (3.3%)
Elevated transaminitis and bilirubin	0	1 (3.3%)	1 (3.3%)
Elevated amylase and lipase	0	1 (3.3%)	1 (3.3%)
Adjuvant phase			
Myocarditis	1(6.25%)	0	1(6.25%)

The postoperative complications were reported in 7 patients (shown in **Supplementary Table 2**) Most comorbidities were mild (Grade 1-2 according to the Clavien-Dindo classification), and the median perioperative hospital stay was 9 days (range 7-23). No patient died within 90 days after surgery.

Out of 16 patients who received postoperative tislelizumab, 12 completed 9 months adjuvant therapy as the study designed. Most didn’t present additional irAEs, but one developed grade 3, histologically confirmed immune-related myocarditis after the 4^th^ cycle of postoperative tislelizumab and recovered after systemic glucocorticoids and tacrolimus.

### 3.5 Biomarker exploration

**Figure 4:**
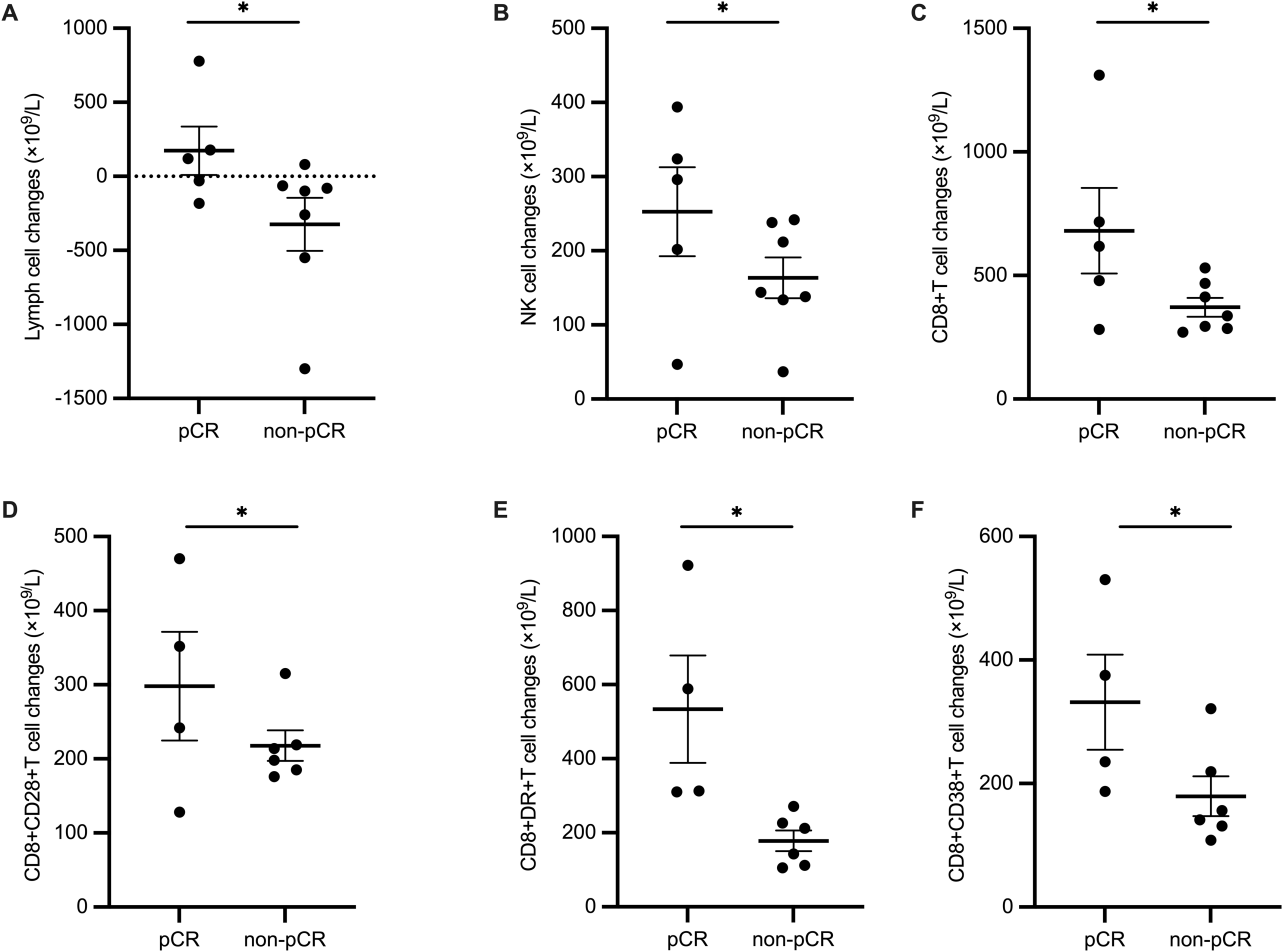
Difference of changes in lymphocyte subsets before and after 2 cycles of neoadjuvant treatment between pCR and non-pCR group. **(A)** Lymph cells, **(B)** NK cells, **(C)** CD8+T cells, **(D)** CD8+CD28+T cells, **(E)** CD8+DR+T cells, and **(F)** CD8+CD38+T cells.

Patients were divided into pCR group and non-pCR group according to whether they achieved pCR using surgically resected tissues. Clinical characteristics were not statistically different between the two groups. (**Supplementary Table 3**) We also explored quantitative lymphocyte subsets in peripheral blood before and during neoadjuvant chemoimmunotherapy for predictors of pathological responses. None of the counts of various lymphocytes at baseline showed statistical differences between pCR and non-pCR group. (**Supplementary Figure 1**) When we analyzed the changes in lymphocyte subsets after 2 cycles of neoadjuvant therapy, we found a group of peripheral lymphocyte subsets increased significantly (P<0.05) in ESCC patients who achieved pCR, including Lymph cells (p=0.042), NK cells, (p=0.042), CD8+T cells(p=0.042), CD8+CD28+T cells(p=0.039), CD8+DR+T cells(p=0.02), and CD8+CD38+T cells(p=0.02). (**Figure 4**, other lymphocyte subsets changes shown in **Supplementary Figure 2**) However, no statistically significant results were achieved when multivariate regression analysis was conducted.

## 4 Discussion

In this phase 2 clinical study, perioperative tislelizumab in combination with neoadjuvant chemotherapy resulted in a pathological complete response (pCR) rate of 44% in patients with potentially resectable esophageal squamous cell carcinoma (ESCC), with a manageable safety profile.

Several studies have explored the application of preoperative chemoimmunotherapy in ESCC and a meta-analysis found that the synthesized pCR rate was 32.9% (14). However, the optimal strategy for neoadjuvant treatment in esophageal cancer has not yet been established. The ESCORT-NEO study utilized a combination of camrelizumab with paclitaxel/albumin-bound paclitaxel and cisplatin (15), while the SIN-ICE study used sintilimab with docetaxel/albumin-bound paclitaxel (16). Some studies have also selected neoadjuvant immunotherapy combined with chemoradiotherapy, achieving good efficacy (17, 18). Based on the data from the JCOG1109 study and our center’s experience, we designed a regimen of 4 cycles of neoadjuvant tislelizumab combined with docetaxel and cisplatin/carboplatin for our study.

Notably, our study was designed to include four cycles of neoadjuvant treatment, more than two cycles used in some other studies. On the one hand, total neoadjuvant therapy (TNT) based on chemotherapy and chemoradiotherapy has been shown to double the pCR rate, reduce distant metastasis, and significantly improve survival in rectal cancer (19). On the other hand, we believe that more cycles of chemoimmunotherapy would better conform to the goals of neoadjuvant treatment for tumor downstaging and micrometastases eradication, as the radiological responses to immunotherapy may be delayed. A small-scale study has suggested that compared to two cycles, four cycles of chemoimmunotherapy as neoadjuvant treatment result in a higher pCR rate and overall response rate (ORR), with no increase in adverse events (20). Interestingly, we conducted a midway assessment after the second cycle using endoscopic ultrasound (EUS) and found that no patients presented with a clinical complete response (CR). However, half of these patients ultimately achieved pCR after four cycles, supporting our viewpoint. Additionally, more than half of the patients in our study were at stage IV, whereas other studies primarily focused on stage III patients (21–23), suggesting that our regimen can achieve significant benefits in patients with more advanced local staging.

In our study, the 6-month and 1-year OS rates were 96.7% and 86.3%. Although the OS and DFS data are immature due to the relatively short follow-up duration, the data in our study showed a trend toward long-term survival benefit which is comparable to previously reported studies. At the 2019 ASCO conference, Hong et al. reported results from a study using pembrolizumab plus concurrent chemoradiotherapy (cCRT) as neoadjuvant treatment in ESCC, with 6-month and 1-year OS rates of 89.3% and 80.8%, respectively (24). Long-term follow-up and randomized studies will provide information on whether adjuvant chemoimmunotherapy will confer comparable survival data to standard nCRT.

Although the treatment cycles were increased, the incidence of severe treatment-related adverse events (TRAEs) in our study was consistent with previous studies, and all events were manageable (16, 22, 25). The incidence of neutropenia was relatively high. However, the use of long-acting granulocyte-colony stimulating factor (G-CSF) as secondary prophylaxis proved to be highly effective, ensuring that no patients were unable to complete the neoadjuvant treatment due to neutropenia. One significant concern during the neoadjuvant treatment phase is that severe adverse events may lead to delays or cancellations of surgery, or even threaten patients’ lives. In our study, only 2 patients experienced delays in surgery due to TRAEs, suggesting that neoadjuvant therapy was overall safe.

Additionally, 10 patients in our study showed elevated serum creatinine levels, which were thought to be caused by multiple factors. 4 patients, after consultation with nephrology experts, were diagnosed with immune-related renal toxicity, and 3 of these diagnoses were confirmed by renal biopsy. The elevated creatinine in the other 6 patients may have been related to cisplatin toxicity or prerenal injury due to inadequate oral intake. This adverse event was unexpected, as the same combination regimen did not induce such a high incidence of acute kidney injury in the treatment of advanced ESCC (9). Although it is difficult to directly attribute the creatinine elevation to the addition of tislelizumab to the cisplatin-based doublet, close monitoring of kidney function and a switch to carboplatin in patients with elevated creatinine levels is warranted when applying our regimen (23, 26–28). Finally, a delayed, grade 3 myocarditis observed in one patient highlights the importance of continuing monitoring for immune-related adverse events (irAEs) in the adjuvant setting.

The negativity of regional lymph nodes is of prognostic significance in locally advanced ESCCs. A prospective study revealed that patients with ypN0/pN0 status after nCRT or direct surgery had superior OS and DFS compared to those with ypN+/pN+ status (29). nCRT has been reported to realize downstaging of lymph nodes in approximately 50% of patients (30). In our study, 80% of patients experienced lymph nodes downstaging. Only 8 out of 25 patients (32%) had residual tumor cells in the lymph nodes (ypN+), and 5 of them recurred early, 4 with distant metastases. Although the number is limited and long-term survival data is immature, our findings may also suggest the sensitivity of regional lymph node involvement at surgery as a potential predictor for the survival in ESCC patients who received neoadjuvant chemoimmunotherapy. Such findings were also observed in NSCLCs, where preoperative chemoimmunotherapy particularly benefited patients with regional lymph nodes metastases (31).

In addition, we discovered that certain peripheral lymphocyte subsets were significantly increased in patients who achieved pCR after 2 courses of neoadjuvant therapy, indicating that the dynamic change of peripheral lymphocyte subsets could become response-predicting biomarkers. These increased subpopulations include Lymph cells, NK cells, CD8+T cells, CD8+CD28+T cells, CD8+DR+T cells, and CD8+CD38+T cells. Previously, a good correlation of immune cell layouts between peripheral blood and tumor tissues was found (32). CD28 mediates the co-stimulatory signal needed by T cell activation and its circulating level was positively associated with the efficacy of PD-1/PD-L1 inhibitors (33). Besides, the expression of HLA-DR+ or CD38+ on CD8+ T cells highlighted pro-inflammatory status in various tumors and predicted excellent efficacy of antitumor treatment including chemotherapy and immunotherapy (34–36). Therefore, it is reasonable to deduct that such circulating lymphocyte subsets, although they can’t be taken for granted as a match to tumor immune microenvironment, can be regarded as a constitutionally activated and inflamed immune phenotype and applied as an early prediction tool for the efficacy of neoadjuvant chemoimmunotherapy in ESCCs.

Our study has some limitations. First, this is a single-arm, phase 2 clinical trial, and further validation of the efficacy in a larger cohort is needed. Randomized studies testing perioperative chemoimmunotherapy versus nCT or nCRT should be carried out. Second, the follow-up period in our study is relatively short, and the DFS and OS results are immature. Therefore, long-term survival benefits of our regimen are waiting to be unveiled. The long-term survival benefits and quality of life outcomes associated with our regimen are waiting to be unveiled. Third, although differences in changes in lymphocyte subsets were found, we failed to find biomarkers that could predict efficacy in multivariate regression due to the small sample size. It’s also a pity that we haven’t yet build a connection between circulating lymphocyte subsets and the authentic tumor immune microenvironment or widely acknowledged efficacy markers of immunotherapy, such as PD-L1 expression level in tumor tissue samples. Further research on molecular and immune landscape is needed to stratify patients and guide individualized perioperative immunotherapy.

## 5 Conclusions

Our study proved that perioperative combinations as neoadjuvant tislelizumab and chemotherapy plus adjuvant tislelizumab obtained promising clinical efficacy with a manageable safety profile in locally advanced ESCCs. This provides compelling evidence for the incorporation of immunotherapy in perioperative treatment for esophageal cancer.

## Data Availability

The raw data supporting the conclusions of this article will be made available by the authors, without undue reservation.
