# Supplementary material for: Perioperative tislelizumab with four cycles of neoadjuvant chemotherapy for resectable locally advanced esophageal squamous cell carcinoma: a phase 2 study
Source: Front Immunol. 2024 Dec 2;15:1482005. doi: 10.3389/fimmu.2024.1482005 (PMC11647006; doi:10.3389/fimmu.2024.1482005)
Supplement: Supplementary file 1 [file DataSheet1.pdf]

Supplementary Table 1. Treatment-related adverse events.

Adverse events, n (%)	Grade 1/2	Grade 3/4	Total
Weakness	3(10%)	0	3(10%)
Nausea	5(16.7%)	0	5(16.7%)
Vomit	0	1(3.3%)	1(3.3%)
Diarrhea	3(10%)	0	3(10%)
Sinus bradycardia	1(3.3%)	0	1(3.3%)
Rash	3(10%)	1(3.3%)	4(13.3%)
Dry skin	1(3.3%)	0	1(3.3%)
Mucositis oral	2(6.7%)	0	2(6.7%)
Lung infection	0	1(3.3%)	1(3.3%)
Anemia	2(6.7%)	0	2(6.7%)
Neutropenia	1(3.3%)	9(30.0%))	10(33.3%)
Creatinine increased	8(26.7%)	2(6.7%)	10(33.3%)
ALT/AST elevated	0	1(3.3%)	1(3.3%)
Bilirubin increased	0	1(3.3%)	1(3.3%)
Pancreatic enzymes elevated	0	1(3.3%)	1(3.3%)
Hypophysitis	1(3.3%)	0	1(3.3%)
Hyperthyroidism	1(3.3%)	0	1(3.3%)
Esophageal fistula	1(3.3%)	0	1(3.3%)

Supplementary Table 2. Perioperative complications.

n=25

Perioperative complications, n (%)	I	II	IIIa	IIIb	IVa
Anastomotic stricture		1(14.29%)			
Broncho-esophageal fistula		1(14.29%)		1(14.29%)	
Fever	1(14.29%)				
Pneumonia		1(14.29%)			
Thrombosis		1(14.29%)			
Hypokalemia	1(14.29%)				

Supplementary Table 3. The differences of baseline clinical characteristics between the pCR group and non-pCR group.

		pCR	non-pCR	P value
n		11	14	
Age (yr), mean (SD)		59.3 (4.6)	61.2 (4.9)	0.326
Sex, n (%)				1
	Female	2 (18.2)	2 (14.3)	
	Male	9 (81.8)	12 (85.7)	
Location, n (%)				0.307
	Upper	3 (27.3)	1 (7.1)	
	Middle	4 (36.4)	4 (28.6)	
	Lower	4 (36.4)	9 (64.3)	
Length (cm), median [IQR]		4.1 [4.0, 7.5]	5.0 [4.0, 6.0]	0.884
T stage, n (%)				1
	T2	1 (9.1)	1 (7.1)	
	T3	6 (54.5)	7 (50.0)	
	T4	4 (36.4)	6 (42.9)	
N stage, n (%)				0.664
	N1	5 (45.5)	5 (35.7)	
	N2	5 (45.5)	5 (35.7)	
	N3	1 (9.1)	4 (28.6)	
TNM Stage, n (%)				0.695

	III	6 (54.5)	6 (42.9)	
	IVA	5 (45.5)	8 (57.1)	
Grade, n (%)				0.919
	G1	1 (9.1)	1 (7.1)	
	G2	7 (63.6)	8 (57.1)	
	G3	1 (9.1)	3 (21.4)	
	Gx	2 (18.2)	2 (14.3)	
Smoke status, n (%)				1
	N	4 (36.4)	4 (28.6)	
	Y	7 (63.6)	10 (71.4)	
Drink status, n (%)				0.288
	N	3 (27.3)	1 (7.1)	
	Y	8 (72.7)	13 (92.9)	

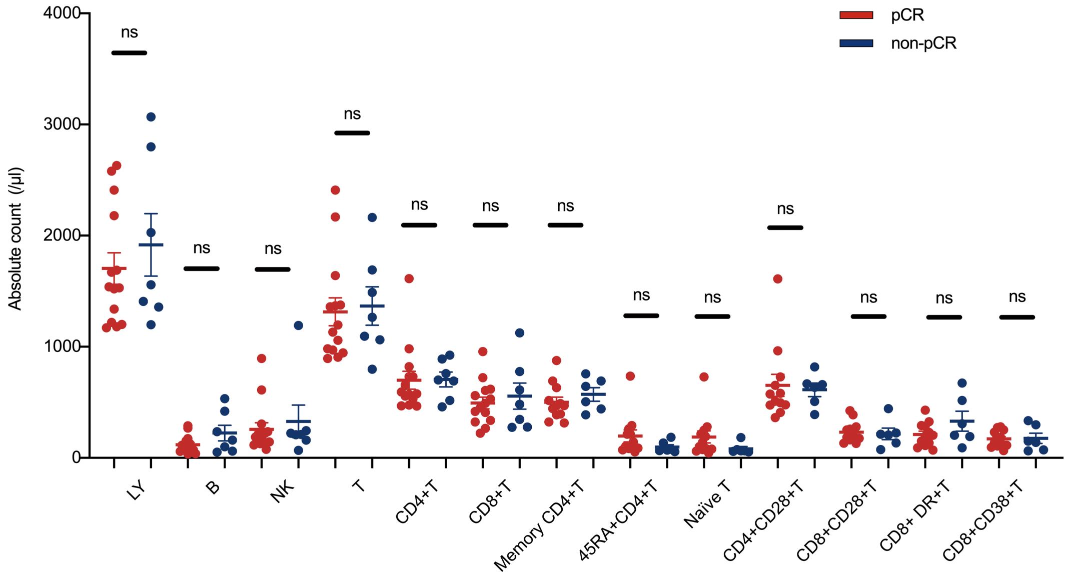

Supplementary Figure 1. The differences of baseline lymphocyte subset between the pCR and non-pCR group.

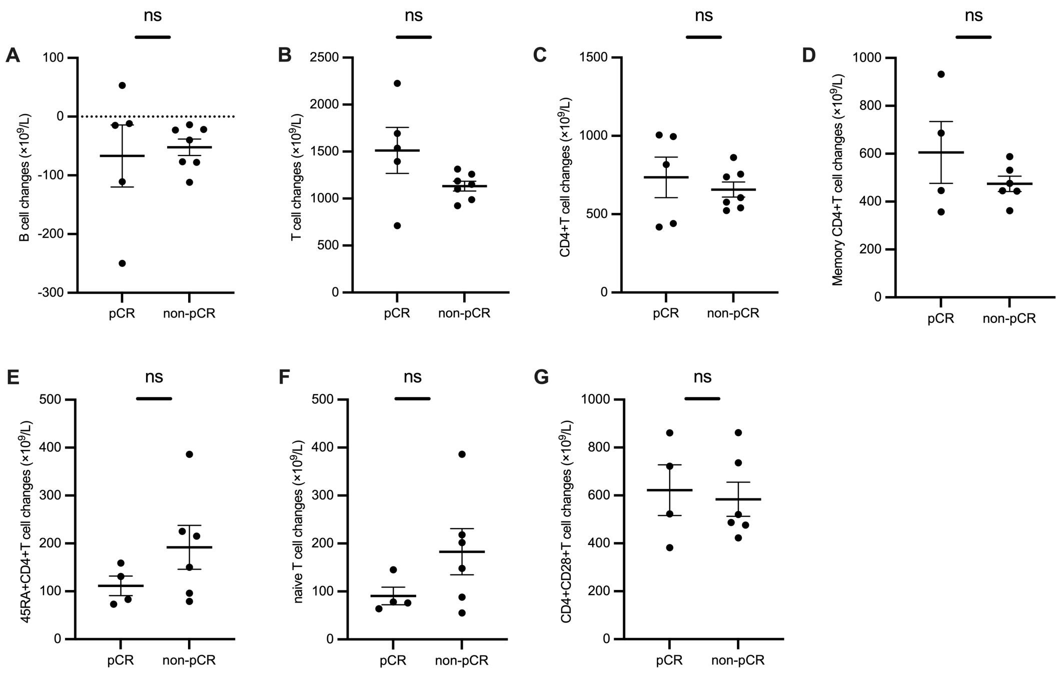

Supplementary Figure 2. The differences of other lymphocyte subset changes after 2 cycles of neoadjuvant treatment between the pCR and non-pCR group.
